# Bis(μ-5-carboxyl­atotetra­zolido)bis­[aqua­(2,2′-bipyrid­yl)cadmium(II)]

**DOI:** 10.1107/S1600536810027613

**Published:** 2010-07-21

**Authors:** Shuang-Jiao Sun, Ji-Hua Deng, Ti-Lou Liu

**Affiliations:** a Shaoyang Medical College, Shaoyang, Hunan 422000, People’s Republic of China; b College of Chemistry and Bio-engineering, Yichun University, Yichun, Jiangxi 336000, People’s Republic of China

## Abstract

In the title dinuclear Cd^II^ complex, [Cd_2_(C_2_N_4_O_2_)_2_(C_10_H_8_N_2_)_2_(H_2_O)_2_], each Cd atom is in a slightly distorted octa­hedral coordination by two N atoms and one O atom of two 1*H*-tetra­zole-5-carboxyl­ate (TZC) ligands, two N atoms of a 2,2′-bipyridyl ligand and one water O atom. The TZC ligand acts in a tridentate *N*,*O*-chelating *N*-bridging mode to two symmetry-equivalent Cd^II^ atoms. The complex reveals mol­ecular *C*_i_ symmetry. Extensive O—H⋯O hydrogen bonding plays an important role in the crystal packing.

## Related literature

For the structural topologies and varied properties such as mol­ecular magnetism, mol­ecular absorption, catalysis, non-linear optics and luminescence of coordination complexes with tetra­zolate-based ligands, see: Zhao *et al.* (2008 ▶); Cheng *et al.* (2007 ▶). For related structures, see: Wu *et al.* (2009 ▶; 2010 ▶) For related literature on 1*H*-tetrazoles, see: Jia *et al.* (2009 ▶); Zhong *et al.* (2010 ▶).
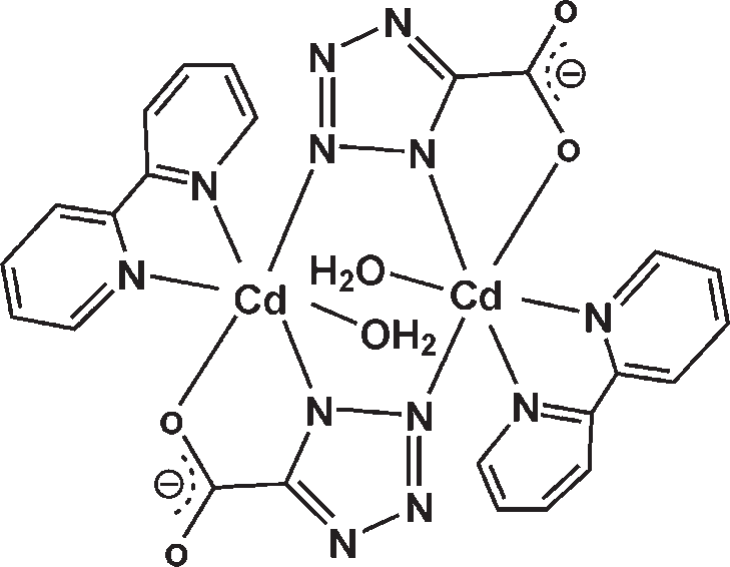


## Experimental

### 

#### Crystal data

[Cd_2_(C_2_N_4_O_2_)_2_(C_10_H_8_N_2_)_2_(H_2_O)_2_]
*M* *_r_* = 797.32
Triclinic, 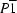

*a* = 7.5218 (13) Å
*b* = 9.6372 (16) Å
*c* = 9.7335 (16) Å
α = 75.628 (3)°
β = 89.686 (3)°
γ = 74.461 (2)°
*V* = 657.10 (19) Å^3^
*Z* = 1
Mo *K*α radiation
μ = 1.69 mm^−1^
*T* = 173 K
0.28 × 0.22 × 0.16 mm

#### Data collection

Bruker SMART CCD area-detector diffractometer
Absorption correction: multi-scan (*SADABS*; Bruker, 1998 ▶) *T*_min_ = 0.650, *T*_max_ = 0.774
3760 measured reflections
2168 independent reflections
1930 reflections with *I* > 2σ(*I*)
*R*_int_ = 0.019

#### Refinement

*R*[*F* ^2^ > 2σ(*F* ^2^)] = 0.025
*wR*(*F* ^2^) = 0.066
*S* = 1.11
2168 reflections
207 parameters
3 restraints
H atoms treated by a mixture of independent and constrained refinement
Δρ_max_ = 0.70 e Å^−3^
Δρ_min_ = −0.47 e Å^−3^

### 

Data collection: *SMART* (Bruker, 1998 ▶); cell refinement: *SAINT* (Bruker, 1998 ▶); data reduction: *SAINT*; program(s) used to solve structure: *SHELXS97* (Sheldrick, 2008 ▶); program(s) used to refine structure: *SHELXL97* (Sheldrick, 2008 ▶); molecular graphics: *SHELXTL* (Sheldrick, 2008 ▶); software used to prepare material for publication: *SHELXTL*.

## Supplementary Material

Crystal structure: contains datablocks I, global. DOI: 10.1107/S1600536810027613/kp2269sup1.cif

Structure factors: contains datablocks I. DOI: 10.1107/S1600536810027613/kp2269Isup2.hkl

Additional supplementary materials:  crystallographic information; 3D view; checkCIF report

## Figures and Tables

**Table 1:** Selected bond lengths (Å)

Cd1—N9	2.285 (3)
Cd1—N2^i^	2.304 (3)
Cd1—N1	2.310 (3)
Cd1—O3	2.314 (3)
Cd1—O1	2.330 (2)
Cd1—N10	2.352 (3)

Symmetry code: (i) 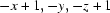.

**Table 2:** Hydrogen-bond geometry (Å, °)

*D*—H⋯*A*	*D*—H	H⋯*A*	*D*⋯*A*	*D*—H⋯*A*
O3—H3*B*⋯O2^ii^	0.83 (2)	2.01 (3)	2.794 (4)	158 (4)
O3—H3*A*⋯O2^iii^	0.80 (2)	2.09 (4)	2.769 (4)	143 (4)

Symmetry codes: (ii) 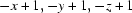; (iii) 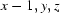.

**Fig. 1.**
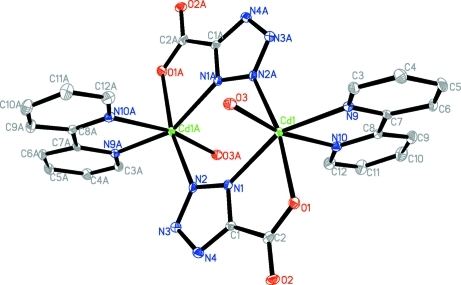
The structure of (I), showing 30% probability displacement ellipsoids and the atom-labeling scheme. Symmetry code: (A) -x + 1, -y, -z + 1.

**Fig. 2.**
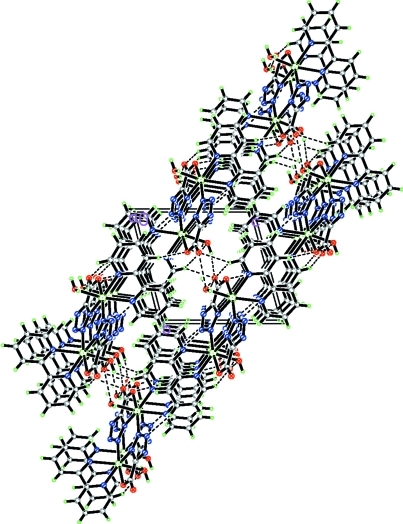
Three-dimensional network of hydrogen bonds.

## supplementary crystallographic information

### Comment

Coordination complexes with tetrazolate-based ligands have been the subject of intense research efforts in recent years, owing to their enormous variety of interesting structural topologies, and wide physical properties such as molecular magnetism, molecular absorption, catalysis, non-linear optics, and luminescence (Zhao, 2008; Cheng *et al.*, 2007). The crystal structures and properties of metal complexes based on tetrazolate-5-carboxylato ligand have been reported in several papers (Wu *et al.*, 2009; Wu *et al.*, 2010) in recent years. Herein, we report the synthesis and crystal structure of its cadmium(II) complex.

In the title compound (I), the asymmetric unit comprises a half of the molecule (Fig. 1) and an inversion symmetry generates a dinuclear complex. The bond lengths (Table 1) and angles around Cd1 atom suggests a slightly distorted octahedral geometry. The TZC ligand acts as a tridentate linker to chelate the Cd atom and bridges the other Cd atom in a µ_2_-N2:O1,N1 coordination mode (Fig. 1). Two Cd atoms are bridged by tetrazolate groups from two symmetry-related TZC ligands to form one six-membered ring (Cd1—N1—N2—Cd1A—N1A—N2A). There are O–H···O, C–H···N, C–H···O intermolecular hydrogen bonds (Table 2). The molecules are held together by intermolecular hydrogen bonding interactions, forming a three-dimensional network (Fig. 2).

### Experimental

A mixture of Cd(NO_3_)_2_ 4H_2_O (0.5 mmol), TZC (0.5 mmol), KOH (0.5 mmol) and 2,2'-bipy (0.5 mmol) in aqueous solution (15 ml) was sealed in a 25 ml Teflon-lined stainless steel vessel under autogenous pressure and heated at 383 K for 3 days, and then slowly cooled to room temperature. Colourless crystals suitable for X-ray analyses were obtained, washed with distilled water and dried in air. Yield: 50% (based on Cd).

### Refinement

The H atoms of the 2,2'-bipy were placed in geometrically idealized positions with C—H distances of 0.93 Å, and were refined isotropic using a riding model with *U*_iso_(H) = 1.2Ueq(C). The H atoms of the coordinated water molecules were assigned in the difference Fourier maps and refined isotropically.

### Crystal data

**Table:**

[Cd_2_(C_2_N_4_O_2_)_2_(C_10_H_8_N_2_)_2_(H_2_O)_2_]	*Z* = 1
*M**_r_* = 797.32	*F*(000) = 392
Triclinic, *P*1	*D*_x_ = 2.015 Mg m^−^^3^
Hall symbol: -P 1	Mo *K*α radiation, λ = 0.71073 Å
*a* = 7.5218 (13) Å	Cell parameters from 2926 reflections
*b* = 9.6372 (16) Å	θ = 2.7–27.0°
*c* = 9.7335 (16) Å	µ = 1.69 mm^−^^1^
α = 75.628 (3)°	*T* = 173 K
β = 89.686 (3)°	Block, colourless
γ = 74.461 (2)°	0.28 × 0.22 × 0.16 mm
*V* = 657.10 (19) Å^3^	

### Data collection

**Table:**

Bruker SMART CCD area-detector diffractometer	2268 independent reflections
Radiation source: fine-focus sealed tube	1930 reflections with *I* > 2σ(*I*)
graphite	*R*_int_ = 0.019
phi and ω scans	θ_max_ = 25.0°, θ_min_ = 2.2°
Absorption correction: multi-scan (*SADABS*; Bruker, 1998)	*h* = −8→8
*T*_min_ = 0.650, *T*_max_ = 0.774	*k* = −11→11
3760 measured reflections	*l* = −11→11

### Refinement

**Table:**

Refinement on *F*^2^	Primary atom site location: structure-invariant direct methods
Least-squares matrix: full	Secondary atom site location: difference Fourier map
*R*[*F*^2^ > 2σ(*F*^2^)] = 0.025	Hydrogen site location: inferred from neighbouring sites
*wR*(*F*^2^) = 0.066	H atoms treated by a mixture of independent and constrained refinement
*S* = 1.11	*w* = 1/[σ^2^(*F*_o_^2^) + (0.0331*P*)^2^ + 0.4626*P*] where *P* = (*F*_o_^2^ + 2*F*_c_^2^)/3
2168 reflections	(Δ/σ)_max_ < 0.001
207 parameters	Δρ_max_ = 0.70 e Å^−^^3^
3 restraints	Δρ_min_ = −0.47 e Å^−^^3^

### Special details

**Table:**

Geometry. All e.s.d.'s (except the e.s.d. in the dihedral angle between two l.s. planes) are estimated using the full covariance matrix. The cell e.s.d.'s are taken into account individually in the estimation of e.s.d.'s in distances, angles and torsion angles; correlations between e.s.d.'s in cell parameters are only used when they are defined by crystal symmetry. An approximate (isotropic) treatment of cell e.s.d.'s is used for estimating e.s.d.'s involving l.s. planes.
Refinement. Refinement of *F*^2^ against ALL reflections. The weighted *R*-factor *wR* and goodness of fit *S* are based on *F*^2^, conventional *R*-factors *R* are based on *F*, with *F* set to zero for negative *F*^2^. The threshold expression of *F*^2^ > σ(*F*^2^) is used only for calculating *R*-factors(gt) *etc*. and is not relevant to the choice of reflections for refinement. *R*-factors based on *F*^2^ are statistically about twice as large as those based on *F*, and *R*- factors based on ALL data will be even larger.

### Fractional atomic coordinates and isotropic or equivalent isotropic displacement parameters (Å^2^)

**Table:**

	*x*	*y*	*z*	*U*_iso_*/*U*_eq_	
Cd1	0.40965 (3)	0.23221 (3)	0.36929 (3)	0.01494 (11)	
O1	0.6019 (3)	0.3838 (3)	0.3780 (3)	0.0205 (6)	
O2	0.8715 (3)	0.3914 (3)	0.4648 (3)	0.0212 (6)	
O3	0.2432 (3)	0.3036 (3)	0.5528 (3)	0.0199 (6)	
N1	0.6687 (4)	0.0890 (3)	0.5152 (3)	0.0145 (6)	
N2	0.7360 (4)	−0.0460 (3)	0.6050 (3)	0.0150 (6)	
N3	0.8896 (4)	−0.0486 (4)	0.6726 (3)	0.0187 (7)	
N4	0.9234 (4)	0.0835 (3)	0.6289 (3)	0.0181 (7)	
N9	0.2429 (4)	0.4286 (3)	0.1938 (3)	0.0151 (6)	
N10	0.5150 (4)	0.1995 (3)	0.1484 (3)	0.0167 (7)	
C1	0.7862 (5)	0.1654 (4)	0.5330 (4)	0.0142 (8)	
C2	0.7527 (5)	0.3265 (4)	0.4521 (4)	0.0174 (8)	
C3	0.1195 (5)	0.5462 (4)	0.2218 (4)	0.0190 (8)	
H3	0.0696	0.5230	0.3173	0.023*	
C4	0.0345 (5)	0.6728 (4)	0.1188 (4)	0.0230 (9)	
H4	−0.0526	0.7532	0.1432	0.028*	
C5	0.0777 (5)	0.6817 (4)	−0.0214 (4)	0.0232 (9)	
H5	0.0196	0.7672	−0.0951	0.028*	
C6	0.2080 (5)	0.5619 (4)	−0.0508 (4)	0.0189 (8)	
H6	0.2427	0.5655	−0.1454	0.023*	
C7	0.2875 (5)	0.4368 (4)	0.0589 (4)	0.0156 (8)	
C8	0.4293 (5)	0.3049 (4)	0.0329 (4)	0.0157 (8)	
C9	0.4693 (5)	0.2928 (5)	−0.1038 (4)	0.0240 (9)	
H9	0.4056	0.3679	−0.1842	0.029*	
C10	0.6035 (6)	0.1695 (5)	−0.1209 (4)	0.0289 (10)	
H10	0.6316	0.1582	−0.2134	0.035*	
C11	0.6960 (6)	0.0634 (5)	−0.0029 (4)	0.0282 (9)	
H11	0.7911	−0.0205	−0.0128	0.034*	
C12	0.6479 (5)	0.0810 (4)	0.1307 (4)	0.0243 (9)	
H12	0.7106	0.0072	0.2123	0.029*	
H3A	0.149 (5)	0.288 (5)	0.531 (5)	0.037 (14)*	
H3B	0.234 (6)	0.388 (4)	0.563 (5)	0.045 (15)*	

### Atomic displacement parameters (Å^2^)

**Table:**

	*U*^11^	*U*^22^	*U*^33^	*U*^12^	*U*^13^	*U*^23^
Cd1	0.01564 (15)	0.01420 (16)	0.01338 (16)	−0.00440 (11)	−0.00143 (10)	−0.00027 (11)
O1	0.0236 (14)	0.0146 (13)	0.0214 (14)	−0.0080 (11)	−0.0036 (11)	0.0020 (11)
O2	0.0216 (13)	0.0211 (14)	0.0258 (15)	−0.0135 (12)	0.0028 (11)	−0.0069 (12)
O3	0.0193 (14)	0.0196 (15)	0.0226 (15)	−0.0068 (12)	0.0009 (11)	−0.0071 (12)
N1	0.0158 (15)	0.0149 (16)	0.0129 (16)	−0.0050 (13)	0.0013 (12)	−0.0028 (13)
N2	0.0163 (15)	0.0139 (16)	0.0122 (16)	−0.0035 (13)	0.0010 (12)	0.0008 (13)
N3	0.0174 (15)	0.0203 (17)	0.0183 (17)	−0.0062 (14)	0.0006 (13)	−0.0037 (14)
N4	0.0158 (15)	0.0193 (17)	0.0186 (17)	−0.0039 (13)	0.0000 (13)	−0.0048 (14)
N9	0.0146 (14)	0.0157 (16)	0.0154 (16)	−0.0054 (13)	0.0004 (12)	−0.0035 (13)
N10	0.0159 (15)	0.0134 (16)	0.0204 (17)	−0.0047 (13)	0.0021 (13)	−0.0027 (13)
C1	0.0130 (16)	0.019 (2)	0.0132 (18)	−0.0056 (15)	0.0047 (14)	−0.0077 (16)
C2	0.0219 (19)	0.022 (2)	0.0133 (19)	−0.0091 (17)	0.0087 (16)	−0.0108 (16)
C3	0.0181 (18)	0.020 (2)	0.017 (2)	−0.0035 (16)	0.0011 (16)	−0.0034 (16)
C4	0.0218 (19)	0.018 (2)	0.025 (2)	−0.0004 (17)	−0.0010 (17)	−0.0043 (17)
C5	0.0231 (19)	0.017 (2)	0.023 (2)	−0.0026 (17)	−0.0066 (17)	0.0033 (17)
C6	0.0233 (19)	0.020 (2)	0.0126 (19)	−0.0088 (17)	0.0002 (16)	0.0000 (16)
C7	0.0150 (17)	0.0170 (19)	0.0151 (19)	−0.0071 (15)	−0.0031 (15)	−0.0018 (16)
C8	0.0146 (17)	0.0166 (19)	0.0159 (19)	−0.0058 (15)	0.0015 (15)	−0.0027 (16)
C9	0.028 (2)	0.027 (2)	0.017 (2)	−0.0085 (18)	0.0041 (17)	−0.0045 (17)
C10	0.034 (2)	0.031 (2)	0.022 (2)	−0.007 (2)	0.0113 (19)	−0.0107 (19)
C11	0.033 (2)	0.018 (2)	0.029 (2)	−0.0001 (18)	0.0075 (19)	−0.0059 (18)
C12	0.024 (2)	0.016 (2)	0.029 (2)	−0.0026 (17)	0.0019 (17)	−0.0024 (17)

### Geometric parameters (Å, °)

**Table:**

Cd1—N9	2.285 (3)	N10—C12	1.349 (5)
Cd1—N2^i^	2.304 (3)	C1—C2	1.510 (5)
Cd1—N1	2.310 (3)	C3—C4	1.372 (5)
Cd1—O3	2.314 (3)	C3—H3	0.9966
Cd1—O1	2.330 (2)	C4—C5	1.388 (6)
Cd1—N10	2.352 (3)	C4—H4	0.9500
O1—C2	1.260 (4)	C5—C6	1.389 (5)
O2—C2	1.242 (4)	C5—H5	0.9500
O3—H3A	0.80 (2)	C6—C7	1.390 (5)
O3—H3B	0.83 (2)	C6—H6	0.9500
N1—C1	1.329 (5)	C7—C8	1.500 (5)
N1—N2	1.342 (4)	C8—C9	1.389 (5)
N2—N3	1.324 (4)	C9—C10	1.382 (5)
N2—Cd1^i^	2.304 (3)	C9—H9	0.9500
N3—N4	1.330 (4)	C10—C11	1.376 (6)
N4—C1	1.330 (5)	C10—H10	0.9500
N9—C7	1.342 (5)	C11—C12	1.386 (6)
N9—C3	1.344 (5)	C11—H11	0.9500
N10—C8	1.342 (5)	C12—H12	0.9500
			
N9—Cd1—N2^i^	106.72 (10)	N4—C1—C2	127.8 (3)
N9—Cd1—N1	155.62 (10)	O2—C2—O1	125.7 (4)
N2^i^—Cd1—N1	96.01 (10)	O2—C2—C1	118.7 (3)
N9—Cd1—O3	95.41 (10)	O1—C2—C1	115.6 (3)
N2^i^—Cd1—O3	87.65 (10)	N9—C3—C4	123.2 (3)
N1—Cd1—O3	94.18 (9)	N9—C3—H3	113.3
N9—Cd1—O1	85.09 (10)	C4—C3—H3	121.4
N2^i^—Cd1—O1	168.16 (9)	C3—C4—C5	119.0 (4)
N1—Cd1—O1	72.19 (10)	C3—C4—H4	120.5
O3—Cd1—O1	92.14 (9)	C5—C4—H4	120.5
N9—Cd1—N10	71.32 (10)	C4—C5—C6	118.2 (4)
N2^i^—Cd1—N10	91.88 (10)	C4—C5—H5	120.9
N1—Cd1—N10	99.78 (10)	C6—C5—H5	120.9
O3—Cd1—N10	166.00 (10)	C5—C6—C7	119.6 (3)
O1—Cd1—N10	91.16 (10)	C5—C6—H6	120.2
C2—O1—Cd1	118.0 (2)	C7—C6—H6	120.2
Cd1—O3—H3A	96 (3)	N9—C7—C6	121.7 (3)
Cd1—O3—H3B	119 (3)	N9—C7—C8	116.5 (3)
H3A—O3—H3B	117 (3)	C6—C7—C8	121.7 (3)
C1—N1—N2	104.9 (3)	N10—C8—C9	121.9 (3)
C1—N1—Cd1	113.4 (2)	N10—C8—C7	116.5 (3)
N2—N1—Cd1	141.1 (2)	C9—C8—C7	121.5 (3)
N3—N2—N1	108.9 (3)	C10—C9—C8	118.8 (4)
N3—N2—Cd1^i^	128.6 (2)	C10—C9—H9	120.6
N1—N2—Cd1^i^	122.5 (2)	C8—C9—H9	120.6
N2—N3—N4	109.4 (3)	C11—C10—C9	119.5 (4)
N3—N4—C1	105.2 (3)	C11—C10—H10	120.2
C7—N9—C3	118.2 (3)	C9—C10—H10	120.2
C7—N9—Cd1	118.7 (2)	C10—C11—C12	118.8 (4)
C3—N9—Cd1	122.4 (2)	C10—C11—H11	120.6
C8—N10—C12	118.8 (3)	C12—C11—H11	120.6
C8—N10—Cd1	116.4 (2)	N10—C12—C11	122.1 (4)
C12—N10—Cd1	124.7 (3)	N10—C12—H12	119.0
N1—C1—N4	111.6 (3)	C11—C12—H12	119.0
N1—C1—C2	120.6 (3)		

Symmetry codes: (i) −*x*+1, −*y*, −*z*+1.

### Hydrogen-bond geometry (Å, °)

**Table:**

*D*—H···*A*	*D*—H	H···*A*	*D*···*A*	*D*—H···*A*
O3—H3B···O2^ii^	0.83 (2)	2.01 (3)	2.794 (4)	158 (4)
O3—H3A···O2^iii^	0.80 (2)	2.09 (4)	2.769 (4)	143 (4)

Symmetry codes: (ii) −*x*+1, −*y*+1, −*z*+1; (iii) *x*−1, *y*, *z*.
